# Variation in diet composition and its relation to gut microbiota in a passerine bird

**DOI:** 10.1038/s41598-022-07672-9

**Published:** 2022-03-08

**Authors:** Lucie Schmiedová, Oldřich Tomášek, Hana Pinkasová, Tomáš Albrecht, Jakub Kreisinger

**Affiliations:** 1 Department of Zoology, Faculty of Sciences, Charles University, Vinicna 7 CZ-128 44, Prague 2, Czech Republic; 2 Institute of Vertebrate Biology, Czech Academy of Sciences, Brno, Czech Republic

**Keywords:** Ecology, Microbiology, Zoology

## Abstract

Quality and quantity of food items consumed has a crucial effect on phenotypes. In addition to direct effects mediated by nutrient resources, an individual’s diet can also affect the phenotype indirectly by altering its gut microbiota, a potent modulator of physiological, immunity and cognitive functions. However, most of our knowledge of diet-microbiota interactions is based on mammalian species, whereas little is still known about these effects in other vertebrates. We developed a metabarcoding procedure based on cytochrome c oxidase I high-throughput amplicon sequencing and applied it to describe diet composition in breeding colonies of an insectivorous bird, the barn swallow (*Hirundo rustica*). To identify putative diet-microbiota associations, we integrated the resulting diet profiles with an existing dataset for faecal microbiota in the same individual. Consistent with previous studies based on macroscopic analysis of diet composition, we found that Diptera, Hemiptera, Coleoptera and Hymenoptera were the dominant dietary components in our population. We revealed pronounced variation in diet consumed during the breeding season, along with significant differences between nearby breeding colonies. In addition, we found no difference in diet composition between adults and juveniles. Finally, our data revealed a correlation between diet and faecal microbiota composition, even after statistical control for environmental factors affecting both diet and microbiota variation. Our study suggests that variation in diet induce slight but significant microbiota changes in a non-mammalian host relying on a narrow spectrum of items consumed.

## Introduction

Diet has a decisive effect on traits tightly linked with fitness, including growth^1^, reproduction^2^, immunity^3^ and various aspects of physiology^4^. At the same time, an animal’s condition and/or health can have a causal effect on the composition of the diet consumed^5^. As an example, the preferred diet may vary with age due to differing nutritional demands related to specific ontogenetic stages^6^.

In addition to the direct consequences of nutrient compounds on fitness-related traits, diet variation may also impose indirect effects by modulating populations of microbial symbionts hosted in the digestive tract of a given individual. This gut microbiota represents the largest fraction of microbial symbionts associated with animal hosts in terms of both cell count and encoded genes^7,8^. Notably, the gut microbiota is a potent modulator of host physiology and health status, with strong effects on the immune system, digestive tract morphology and digestion efficiency. At the same time, disruptions to the normal gut microbiota have been associated with a number of adverse consequences to host health^9,10^.

Diet composition has been identified as one of the main drivers of gut microbiota variation in mammals. At larger phylogenetic scales, for example, repeated transitions between carnivory and herbivory were followed by consistent changes in gut microbiota content^11–13^. In omnivorous species, including humans, both long-term and short-term dietary habits impose gut microbiota changes that partly recapitulate transitions observed at the herbivore-carnivore continuum^14–16^. On the other hand, the effect of diet on gut microbiota has also been observed in species relying on a relatively narrow diet spectrum. For example, significant gut microbiota differences have been detected between folivore and frugivore lemurs^17^, in bison fed on pasture or a grain diet^18^ and even between strictly insectivorous bat species differing in consumed prey^19^.

In comparison with mammals, bird diet appears to be a less important factor modulating gut microbiota^20^. Most studies undertaken on birds have found some support for the effect of diet on interspecific variation, particularly in passerines^21–24^. However, many of these studies had limitations related to the use of indirect dietary data and dietary data based on literature searches, but see^25–27^. A rather high within-species variation in dietary items consumed in some cases could mean that an important source of variation was omitted from these analyses. Consequently, further research of within-species diet variation may improve our understanding of dietary-induced gut microbiota changes in free-living populations.

Analyses of diet in free-living populations are usually based on macroscopic examination of faecal samples or undigested food remains^28,29^. However, this approach is time consuming and demanding in terms of a researcher's expertise. Moreover, there may be a non-negligible risk of limited taxonomic resolution or other specific biases^30,31^. Stable isotopes analysis represents complementary macroscopic method that has provided important insights into the foraging ecology of free-living populations. This technique has also been successfully used to study the effects of diet on the gut microbiota^26,32,33^. On the other hand, information on the taxonomic composition of the ingested diet based on the stable isotopes approach is limited. DNA-based methodologies such as metabarcoding, which rely on deep sequencing of DNA markers bearing taxonomic information, represent a promising alternative that could partly overcome such challenges^34–36^. On the other hand, there are some concerns with metabarcoding, such as the poor amplification of certain taxa and/or the weak correlation between their biomass and the corresponding proportions of the sequences^37,38^.

Studies on diet-microbiota interactions in wild populations of birds are still rare, probably due to scarcity of data on interindividual variation in diet. To our knowledge, there is only one study exploring the effects of natural within-species diet variation on gut microbiota content in birds^39^. Furthermore, Teyssier et al.^40^ demonstrated the effect of diet on intraspecific gut microbiota variation in an omnivorous passerine bird through experimentally induced dietary changes. In this study, we developed procedures for metabarcoding-based diet profiling in insectivorous birds and applied this approach in studying interactions between diet and gut microbiota in breeding population of a migratory passerine bird, the barn swallow (*Hirundo rustica*). In the first step, we explored potential drivers of diet composition in the barn swallow, e.g. temporal variation during the course of the breeding season, spatial variation between breeding colonies and variation between adults and their nestlings. As a further step, we combined existing data on barn swallow faecal microbiota^41^ with diet profiles for the same individuals to test whether interindividual variation in diet was a predictor of gut microbiota composition and its predicted functions. To our knowledge, our contribution represents the first attempt to integrate individual-based data on metabarcoding-based diet and faecal microbiota composition in an insectivorous bird.

## Material and methods

### Sample collection

We used previously extracted metagenomics DNA samples collected for our previous study on the faecal microbiota of barn swallows^41^. Collection of faecal samples from both adults and nestlings (6–12 days after hatching) was conducted at two colonies (Šaloun farm, Lomnice nad Lužnicí [49° 4′ 7.762″ N, 14° 42′ 36.521″ E]; Hamr farm, Lužnice [49° 3′ 25.288″ N, 14° 46′ 10.82″ E]) in the Třeboňsko Protected Landscape Area (Czech Republic; distance between populations = 4.5 km) during the barn swallow breeding season from May to August 2014. The average Julian date of sampling did not differ between the two sites (Welsh t-test: d.f. = 79.9, t = 1.329, p = 0.1843). Both farms are located in a landscape dominated by intensive agriculture and consisted of a mosaic of agricultural fields, hay meadows, fish ponds, and small secondary coniferous or mixed forest patches. The farms differed in terms of the species farmed, with sheep and goats being more prevalent in Šaloun, while production in Hamr farm was more focused on cows and pigs.

To collect faecal samples, adults were placed in a paper bag and nestlings in a plastic beaker filled with paper towels, where they were kept for approx.30 min. Faeces were harvested using a sterile microbiological swab (Copan, Italy), placed in sterile DNA/RNA free cryotubes (Simport, Canada) and stored in liquid nitrogen or at −80 °C for further laboratory analysis. For details on field procedures and faecal sample collection see Kreisinger et al.^41^ and Petrželková et al.^42^. Only a single sample was analysed for each individual. We included 140 individuals (47 adults and 93 juveniles) in this study. However, as explained later, we were able to generate useful sequencing data only for 82 individuals (17 adults and 65juveniles), which were included in the final analyses (Supporting information Table A1).

### Laboratory analysis

Metagenomic DNA from faecal samples was extracted using commercial PowerSoil kits (MoBio), with faecal microbiota subsequently profiled through high-throughput sequencing of 16S rRNA amplicons, as described in our previous studies^41,43^. In brief, the V3-V4 variable regions of 16S rRNA were amplified through a polymerase chain reaction (PCR) using universal primers S-D-Bact-0341-b-S-17 (CCTACGGGNGGCWGCAG) and S-D-Bact-0785-a-A-21 (GACTACHVGGGTATCTAATCC)^44^. Next, sequencing libraries were prepared using TruSeq nano kits (Illumina) and sequenced on Illumina Miseq using the v3 kit (300 bp paired-end reads) at Montpellier-SupAgro (France).

For the purpose of diet profiling, we used universal Cytochrome c oxidase subunit I (COI) primers (BF2-GCHCCHGAYATRGCHTTYCC and BR2-TCDGGRTGNCCRAARAAYCA) targeting a broad range of invertebrate taxa^45^. We selected these primers as previous in vitro and in silico tests indicated that these primers exhibit a low level of PCR bias compared to existing alternatives^45^.

To reduce problems associated with the formation of primer-dimers, sequencing libraries were prepared in three PCR steps:

1. COI pre-amplification by gene-specific primers, using a PCR mixture consisting of 5 µl of PCR mastermix, 0.6 µM of forward and reverse COI-specific primer and 3.8 µl of metagenomic DNA. Our pilot PCR analysis revealed that the primers showed a strong affinity to the host DNA. To avoid amplification of host COI, 6 µM of a custom blocking primer containing C3 spacer modification on the 3' end and exhibiting a perfect match to the host COI (ACCGAAGAACCAGAATAGGTGTTGGTAAAGTAC) was added to the PCR reaction. To evaluate potential biases associated with this technique, a subset of samples (n = 23) was also amplified without the blocking primer. PCR cycling conditions consisted of an initial denaturation step (95 °C, 5 min) followed by 22 cycles of denaturation (98 °C, 20 s), blocking primer annealing (53 °C, 15 s), COI-specific primer annealing (47 °C, 15 s) and extension (72 °C, 40 s), followed by a final extension at 72 °C for 5 min.
2. Amplification by primers including tails compatible with sequencing adaptors, using a PCR mixture comprising 5 µl of PCR mastermix, 2.8 µl of ddH_2_O, 0.6 µM of forward and reverse COI primers flanked by tails complementary to Access Array sequencing adaptors (Fluidigm Corporation, USA) and 1 µl of PCR product from the 1st PCR round. PCR cycling conditions comprised an initial denaturation step (95 °C, 5 min) followed by 15 cycles of denaturation (98 °C, 20 s), primer annealing (50.5 °C, 15 s) and extension (72 °C, 40 s), followed by a final extension at 72 °C for 5 min.
3. PCR-based ligation of sequencing adaptors, using a reaction mixture comprising Access Array sequencing adaptors (4 µl) along with PCR mastermix (10 µl), 4 µl of ddH_2_O and 2 µl of 25 × diluted PCR product from the 2nd PCR round. PCR cycling consisted of an initial denaturation step (95 °C, 5 min) followed by 16 cycles of denaturation (98 °C, 20 s), primer annealing (55.5 °C, 20 s), and extension (72 °C, 40 s), followed by a final extension at 72 °C for 5 min.

Kappa HIFI HotStart polymerase mastermix (Kapa Biosystems, USA) was used in all PCR reactions. Technical PCR duplicates were prepared for all samples. Products from the 3rd PCR round were quantified by GenoSoft software (VWR International, Belgium) based on band intensities after electrophoresis on 1.5% agarose gel and mixed at equimolar concentration. The final library was purified using Agencourt AmpureXP beads (Beckman Coulter Life Sciences). Products of the desired size were extracted by PipinPrep (Sage Science Inc., USA) and sequenced on Illumina Miseq (v3 kit, 300 bp paired-end reads) at the Central European Institute of Technology (CEITEC, Masaryk University, Brno, Czech Republic).

### Bioinformatic analysis of diet profiles

Regions corresponding to gene-specific primers were removed from fastq files using skewer^46^. Subsequently, the fastq files were quality-filtered (< 2 expected error per read) and denoised using R version 3.4.4^47^, with the dada2 package^48^ used to define reliable COI amplicon sequence variants (ASVs). Technical duplicates showed significant consistency in Shannon diversities (Pearson correlation: r = 0.982, p < 0.0001) and composition of COI profiles (Procrustean analysis: r = 0.996, p < 0.0001). Consequently, we merged COI profiles for sample duplicates to obtain sample-specific COI profiles. To suppress any effect of PCR and sequencing artefacts, ASVs that were not consistently present in both technical duplicates were eliminated from the dataset e.g.,^49^. For a limited number of samples, we failed to sequence both duplicates (n = 3). In these cases, we eliminated all ASVs whose presence was not confirmed in samples for which both duplicates were available.

For the purpose of taxonomic classification, 200 top blastn hits for each COI ASV were downloaded from the NCBI nt database and used for the construction of a reference database. Dada2 implementation of RDP classifier^50^ was subsequently applied for taxonomic assignment of COI ASVs at an 80% posterior confidence threshold. Abundances matrix, representing read counts for individual ASVs in each sample, along with sample metadata, taxonomic annotations and ASVs sequences were merged into a phyloseq database^51^.

### Bioinformatic analysis of faecal microbiota

To assess the effect of diet on faecal microbiota, we used sequencing data previously published in Kreisinger et al.^41^. The steps for quality filtering, data denoising, and ASV frequency matrix generation were the same as described above. Chimeric ASVs were detected and eliminated using UCHIME^52^ and the gold.fna reference (available at https://drive5.com/uchime/gold.fa). The taxonomy of non-chimeric ASVs was assigned using the RDP classifier^50^ and the Silva database (version 138)^53^ as a reference. We also excluded ASVs corresponding to mitochondria, chloroplasts, or those that were not assigned to any bacterial phylum. Similarly to diet profiling, technical PCR duplicates were made for each faecal microbiota sample. We checked the consistency of their content using Procrustean analysis and eliminated ASVs that were not consistently present in both technical duplicates. Finally, the sequences of the ASVs were aligned using R package DECIPHER^54^ and a phylogenetic tree was constructed using FastTree2^55^.

Bacterial metagenome functional predictions were conducted using PICRUSt2 pipeline^56^ using default setup, and predicted metagenomes were categorized into MetaCyc pathways^57^. Their predicted abundances were used in later statistical analyses.

### Statistical analyses

Krona pie-charts^58^ were used to visualise the taxonomic content of the whole COI dataset. Next, all non-insect ASVs (i.e. not corresponding to putative dietary items) were eliminated. Congruence in Shannon diversities (calculated after the exclusion of non-insect ASVs; hereinafter termed dietary profile) between sample pairs amplified either with or without the blocking primer were assessed as intra-class correlations calculated using the rptR function in the R statistical environment assuming Gaussian error distribution^59^. We also evaluated congruence in the composition of insect dietary profiles using Procrustean analysis, with Hellinger dissimilarity matrices scaled by Principal Coordinate Analysis (PCoA) used as inputs.

Analysis of covariance (ANCOVA) was used to test whether Shannon diversity (square-root-transformed) of dietary profiles was affected by locality, Julian date of sample collection, age class (i.e. adult vs. young) and by two-way interactions between these variables. Julian date was centred^60^, both in this statistical model and all later analyses. We also checked whether diversity varied with sequencing depth (log-scaled). Significant predictors of dietary diversity were identified via step-wise backward elimination of nonsignificant variables from the initial full model (i.e. containing all the above-mentioned predictors). After visual exploration of divergence in dietary profile composition by PCoA, variation in dietary composition due to the effect of locality, age class and Julian date of sample collection was analysed by distance-based redundancy analysis (db-RDA)^61^ running on Hellinger and binary Jaccard dissimilarities among samples. We considered linear, quadratic and cubic effect of Julian date to account for potentially non-monotonic abundance changes in dietary items during the breeding season. Hellinger dissimilarities automatically account for the different number of sequences between samples. Jaccard dissimilarities were calculated after rarefaction of the abundance matrix (n = 536 sequences per sample, i.e. the minimum sequencing depth achieved). To demonstrate that rarefaction has a negligible effect on overall beta diversity, we calculated Jaccard dissimilarities for a subset of samples with > 5000 sequences (n = 47), which were rarefied to either 500 or 5000 seqs./sample. The resulting strength of correlation between these two distance matrices was high (Procrustean analysis: r = 0.934, p = 0.0001). The db-RDA model selection strategy was based on the forward step-wise approach implemented in the ordiR2step function (vegan package in R)^62^. The abundances of dietary taxa that varied due to the effects of predictors suggested by db-RDA were identified using generalised linear models with negative binomial distribution in the DESeq2 package^63^. False discovery rates (FDR)^64^ were used for multiple testing corrections.

To examine beta diversity of the faecal microbiota, we used phylogenetically controlled weighted and unweighted UniFrac distances in addition to Jaccard and Hellinger dissimilarities. UniFrac only marginally accounts for variation caused by phylogenetically related ASVs. The unweighted UniFrac, which only accounts for the absence/presence of ASVs, was calculated after the abundance matrix rarefaction (threshold = 2315 sequences per sample). The weighted UniFrac (accounting for variation in the abundance of ASVs) was calculated based on the proportions of ASVs in each sample. To infer variation in predicted metagenome content, only Hellinger dissimilarities in functional profiles were used. Procrustean analysis was applied to test for correlation between interindividual divergence in diet and faecal microbiota or predicted metagenome profiles. Furthermore, we employed db-RDA and variation partitioning analysis (varpart function in the R package vegan) to account for direct and indirect effects of environmental factors, where dissimilarities in faecal microbiota composition (i.e. Hellinger , Jaccard, or UniFrac) or predicted metagenomes (only Hellinger dissimilarities) were considered as a response and divergence in diet composition (i.e. PCoA axis scores for Hellinger or Jaccard divergences in diet profiles) and matrix of other variables (including linear, quadratic and cubic effects of Julian date, locality and age class) were included as explanatory and/or conditional variables. To prevent db-RDA model overfitting, we only considered PCoA axes for diet that exhibited a significant correlation with microbiota composition, selected using a forward selection approach (ordiR2step function from R package vegan). Finally, we applied the joint species distribution model (JSDM) from the boral package^65^ to estimate pair-wise residual correlations between diet taxa and bacterial ASVs of predicted metagenome pathways after accounting for the effects of explanatory variables. A similar approach was previously applied to search for cross-domain correlations between faecal microbiota and the intestinal helminth community^66^. To run JSDM, we merged community matrices for insect genera and 16S rRNA ASVs or predicted metagenome profiles and used them as model responses. To account for uneven sequencing depth, we used a model offset equal to the log-transformed number of sequences for a given sample and marker gene (or total predicted abundance of all metagenomic pathways in given sample). Dietary genera and bacterial ASVs detected in < 10 samples were excluded. Similarly, metagenome features with a relative abundance < 0.01% were not considered. Locality, age class and Julian date of sample collection were considered as explanatory variables. We considered JSDM versions, with the effect of Julian date modelled as either, a linear and quadratic term, or a linear, quadratic and cubic term and reported residual correlations that received substantial support based on 95% posterior credible intervals for both these JSDM versions. The models were fitted using default priors (described in boral documentation) and assuming negative binomial distribution of read counts for bacterial and dietary taxa. Diet vs. microbiota taxa correlations were estimated based on a Markov Chain Monte Carlo simulation consisting of 50,000 iterations. The thinning interval was set to 40 iterations, with the first 1000 iterations discharged as burn-in.

### Ethics declarations

All field procedures were conducted in accordance with European Union Guidelines for Animal Care and Treatment and approved by the Animal Care and Use Committees at the Czech Academy of Sciences (041/2011) and Charles University in Prague (4789/2008-0).

## Results

### Effect of blocking primers on COI amplification

We analysed faecal samples from 140 individuals (47 adults and 93 juveniles). PCR amplification failed in 34 samples; hence, we only sequenced 106 individuals (23 adults and 83 juveniles) for the COI profile. We obtained 2,369,181 high-quality reads that were grouped into 1,591 COI ASVs. Median sequencing depth corresponded to 13 258 sequences per sample (range = 536–60,484). Insects representing putative dietary components formed the dominant fraction of COI profiles (47% of all reads, 961 ASVs). Non-target taxa were represented by avian ASVs (18% of reads), plants (10% of reads), fungi (namely Oomycetes, 3% of reads) and putative symbiotic Arachnida (Trombidiformes and Dermanyssidae, 6% reads; Supporting information Fig. A1). The relative abundance of avian ASVs was significantly higher when blocking primer was not included in the first PCR reaction (62% of reads per sample vs. 1.3% of reads; Wilcoxon rank sum test: W = 11, p < 0.0001).

**Figure 1:**
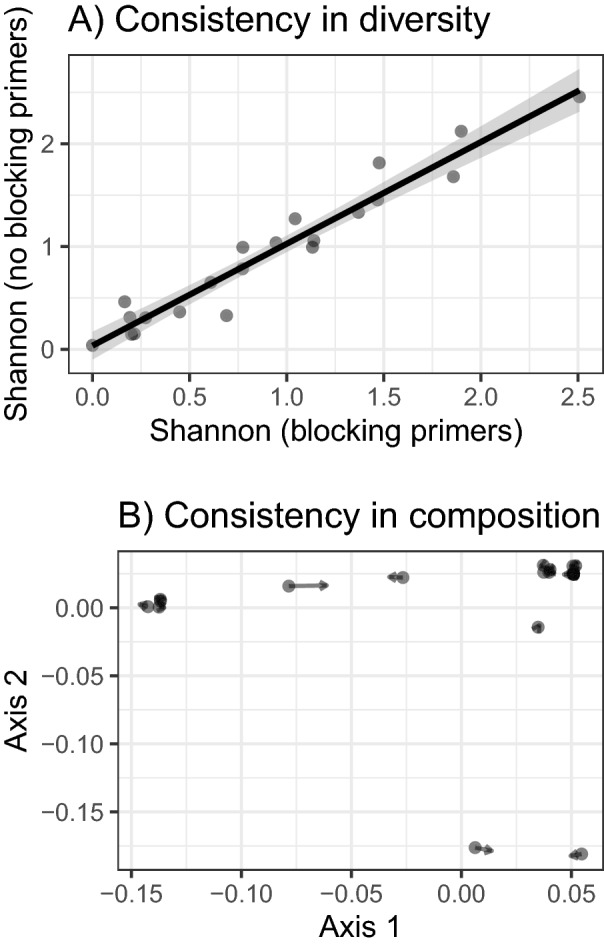
Consistence between diet profiles generated with or without blocking primer, assessed based on (**A**) Shannon diversity correlations and (**B**) procrustean superimposition for Hellinger dissimilarities in diet profiles.

Next, we evaluated the potential effects of using the blocking primer on alpha diversity and composition of dietary profiles (i.e., insect ASVs only). Because the number of insect reads was very low for a subset of the samples amplified without blocking primer, we performed these comparisons for 21 pairs of samples, both of which contained at least 400 insect reads. Sample pairs that were amplified both with and without blocking primers (n = 21 pairs) exhibited high consistency in Shannon diversities (Intra-class correlation = 0.961, 95% bootstrap confidence intervals = 0.927–0.987, permutation-based p = 0.0001) and relative abundance of individual ASVs (Procrustean analysis: r = 0.999, p < 0.0001; Fig. 1). For the purpose of all subsequent analyses, diet profiles for sample duplicates generated with and without blocking primers were merged and insect ASVs were grouped into genus-level bins.

### Diet profile variation

Because the total number of insect reads was low for a subset of individuals, which could negatively affect the robustness of dietary profiles, we based all analyses on diet variation and faecal microbiota-diet correlations on 82 individuals (17 adults and 68 juveniles; Supporting information Table A1) with > 400 insect reads, while 24 individuals with < 400 insect reads were excluded. Importantly, our later missing data analyses suggest that the excluded samples with small numbers of insect reads did not differ in diet composition.

**Figure 2:**
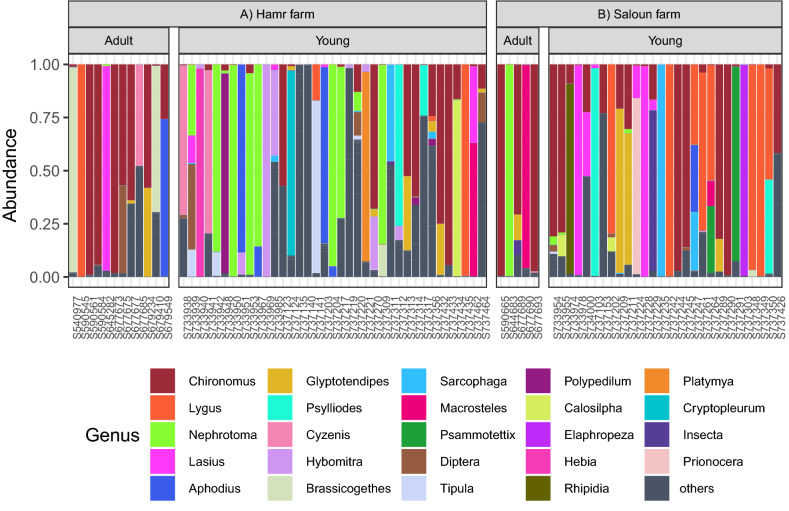
Insect genera detected by diet profiling of barn swallow faecal samples. The average proportion of reads is shown. Taxa present at low abundances (< 1% of all reads in the entire dataset) are indicated as "others".

We detected 171 insect genera or higher insect taxa (in the case of insufficient support for genus-level delimitation). In terms of reads counts, the most abundant insect order was Diptera (61% of reads per sample on average, dominated by the genera *Chironomus* and *Nephrotoma*). A considerable proportion of the dietary profile comprised Hemiptera (17% of reads, dominated by the genus *Lygus*), Coleoptera (14% of reads, dominated by the genera *Aphodius* and *Psylliodes*) and Hymenoptera (6% of reads, dominated by ants of the genus *Lasius*; Supporting information Fig. A2). Other taxa were represented by < 1% reads per sample on average. The number of insect genera per sample ranged between one and 18 (median = 4, mean = 5.22). Individual samples were mostly dominated by a single insect genus (Fig. 2). Subsequently, rarefaction analysis for 1–5001 randomly selected reads per sample revealed that sequencing coverage corresponding to ~ 500 sequences per sample was sufficient to capture the majority of genus-level diversity (Supporting information Fig. A3).

Using ANCOVA, we found that the Shannon diversity of dietary profiles differed between localities (F_(1,80)_ = 5.087, p = 0.0268). However, there was no difference in diversity between adults and juveniles (F_(1,79)_ = 1.831, p = 0.180, mean Shannon diversity [± S.E] = 0.4047 ± 0.087 for adults and 0.663 ± 0.065 for juveniles) and we found no support for any other predictor (i.e. by Julian date of sample collection and by two-way interactions between predictors) of diet diversity (p > 0.05 in all cases).

**Figure 3:**
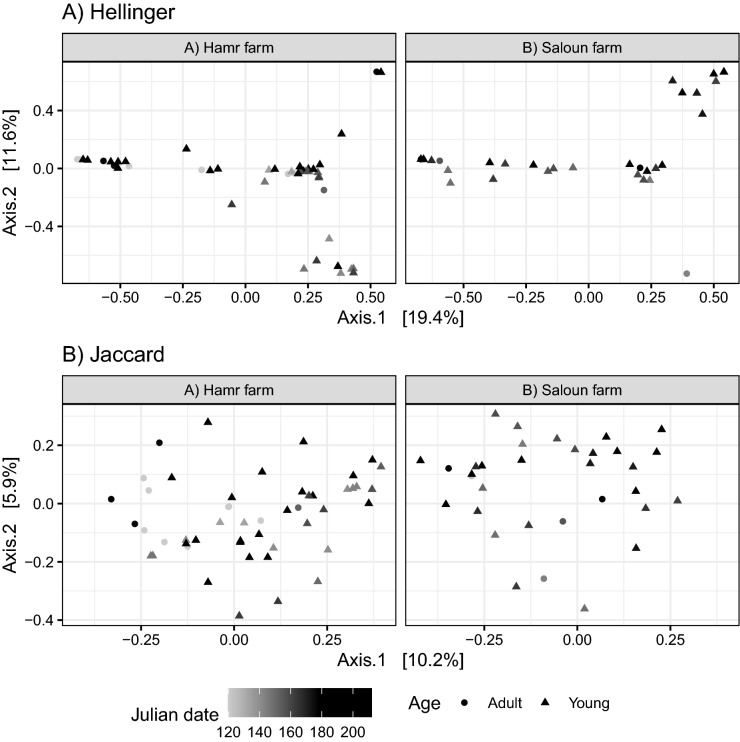
Variation in diet profile composition based on PCoA running on (**A**) Hellinger and (**B**) Jaccard dissimilarities. Samples from adult versus young are indicated by different plotting characters. Samples taken during the breeding season are indicated with different shades of grey. Data for different localities are in different facets.

Explorative insights provided by PCoA for Jaccard and Hellinger dissimilarities suggested an effect of both locality and Julian date on variation in dietary composition (Fig. 3). Specifically, scores for the second PCoA axis separated samples from different localities (Wilcoxon test: W = 459, p < 0.0001 for Jaccard and W = 514, p = 0.005 for Hellinger dissimilarities) and were correlated with Julian date of sample collection (Spearman correlation, rho = 0.248, p = 0.025 for Jaccard and rho = 0.413, p = 0.0001 for Hellinger dissimilarities). Constrained db-RDA models running on Hellinger and Jaccard dissimilarities provided comparable results (Table 1). However, neither PCoA nor db-RDA supported a difference in dietary composition between adults and juveniles. Inclusion of polynomial terms into the final db-RDA models suggested non-monotonic variation in dietary items during the breeding season. Subsequently, DESeq2 analysis aimed at identifying particular insect genera involved in this variation included both the effect of locality and Julian date. In the case of Julian date, we tested for the effect of cubic and quadratic polynomes via likelihood ratio tests. While no insect genera exhibited cubic association with Julian date of sample collection, the abundance of 14 insect genera exhibited quadratic correlation with sampling date (Supporting information Fig. A4 and Table A2). For example, flies from the genera *Pollenia* and *Hybomitra* and from the family Tabaninae, as well as crane flies (genus *Nephrotoma*) and ants (genus *Lasius*), were most commonly detected in the middle of the breeding season. Conversely, beetles from the genus *Aphodius* were more common at the beginning of the breeding season, while mosquitoes from the genera *Culiseta* and *Ochlerotatus*, as well as Hemiptera from the genus *Lygus*, were more prevalent late in the breeding season. No insect genus exhibited significant variation between localities after statistical control for within-season variation (i.e. quadratic effect of sample collection date) and multiple testing corrections.

**Table 1:** ANOVA table for db-RDA models testing the effect of Julian date, locality and age class on variation in the composition of insect profiles. The matrix of A) Hellinger or B) Jaccard dissimilarity in insect profile composition was used as a response. Models were constructed using the forward selection process (ordiR2step function from the R package vegan).

Dissimilarity	Predictor	Df	Variance	F	P
Hellinger	Julian date (linear effect)	1	0.018	1.853	0.032
	Julian date (quadratic effect)	1	0.020	2.029	0.010
	Julian date (cubic effect)	1	0.033	3.297	0.001
	Locality	1	0.020	2.066	0.011
	Residual	77	0.763		
Jaccard	Julian date (linear effect)	1	0.011	2.126	0.001
	Julian date (quadratic effect)	1	0.010	2.012	0.003
	Julian date (cubic effect)	1	0.011	2.114	0.005
	Locality	1	0.011	2.235	0.004
	Residual	77	0.389		

### Missing data analyses

As already mentioned, we excluded samples in which PCR failed (n = 34) and in which the number of target insect reads was low (n = 24). Here we investigated the reasons for the relatively high frequency of these failures, as their non-random distribution could potentially influence interpretations of diet variation and diet-microbiota correlation analyses.

We found that PCR failure was more common in adults (51.1%) than in juveniles (10.8% chi-squared test: d.f. = 1, χ^2^ = 27.591, p < 0.0001). Based on electrophoresis gel band intensity, we observed lower PCR outputs for bacterial 16S rRNA amplicons in adults than in juveniles (Welsh t-test: d.f. = 124.44, t = − 3.458, p = 0.001; amplicons prepared in parallel for the same samples). Hence, we suggest that the lower PCR success in adults was caused by an overall lower quantity and quality of DNA template, probably due to lower amount of faecal material (Schmiedova et al. unpublished). On the other hand, we did not detect any effect of sample location (chi-squared test: d.f. = 1, χ^2^ = 0.037, p = 0.848) or Julian date (Welsh t-test: d.f. = 45.882, t = 0.315, p = 0.755) on the probability of PCR failure. In addition, we found no difference in microbiota composition between samples that failed vs. those that passed the PCR step with diet primers (PERMANOVA: pseudo-F_(1,137)_ = 0.9156, R^2^ = 0.006, p = 0.459 for weighted UniFrac and pseudo-F_(1,137)_ = 1.1648, R^2^ = 0.008, p = 0.157 for Hellinger dissimilarities) after statistical control for the effect of age and locality (i.e. predictors that impacted microbiota composition in our population)^41^.

Frequency of samples with a low vs. sufficient number of insect reads did not vary between adults and juveniles (chi-squared test: d.f. = 1, χ^2^ = 0.199, p = 0.656) or Julian date (Welsh t test: d.f. = 24.088, t = − 0.550, p = 0.588). However, there was a higher percentage of samples with a low number of insect reads at Saloun farm (40%) than Hamr farm (4%; chi-squared test: d.f. = 1, χ^2^ = 19.666, p < 0.0001), which was paralleled by a higher fraction of no-target reads at Saloun farm (58.8%) than at Hamr farm (31.3%) across all sequenced samples (Welsh t test: d.f. = 103.75, t = 3.904, p = 0.0002). This difference was mainly associated with increased percentage of symbiotic Arachnida (36.9% vs. 4.3% of reads) at the former location, suggesting that higher abundance of these nontarget taxa could compromise efficient amplification of insect DNA. Alternatively, it could also be that insect taxa that were poorly captured by our wet-lab protocol were more abundant at Saloun Farm. It is likely that this difference would affect resulting PCR yields. Contrary to this prediction, the concentration of PCR products, as determined by the gel band intensities of the gel bands for diet amplicons, was the same at both sites (Welsh t test: d.f. = 103.57, t = − 0.446, p = 0.656). Importantly, we also found no difference in microbiota composition (PERMANOVA : pseudo-F_(1,103)_ = 0.969, R^2^ = 0.010, p = 0.284 for weighted UniFrac and pseudo-F_(1,103)_ = 0.904, R^2^ = 0.009, p = 0.498 Hellinger distances) or diet composition (PERMANOVA for Hellinger distances: pseudo-F_(1,101)_ = 1.052, R^2^ = 0,01, p = 0.376, only samples with at least one insect read) between samples with low versus sufficient number of insect reads while we accounted for locality-specific variation in microbiota or diet content.

### Association between diet variation and faecal microbiota composition

**Figure 4:**
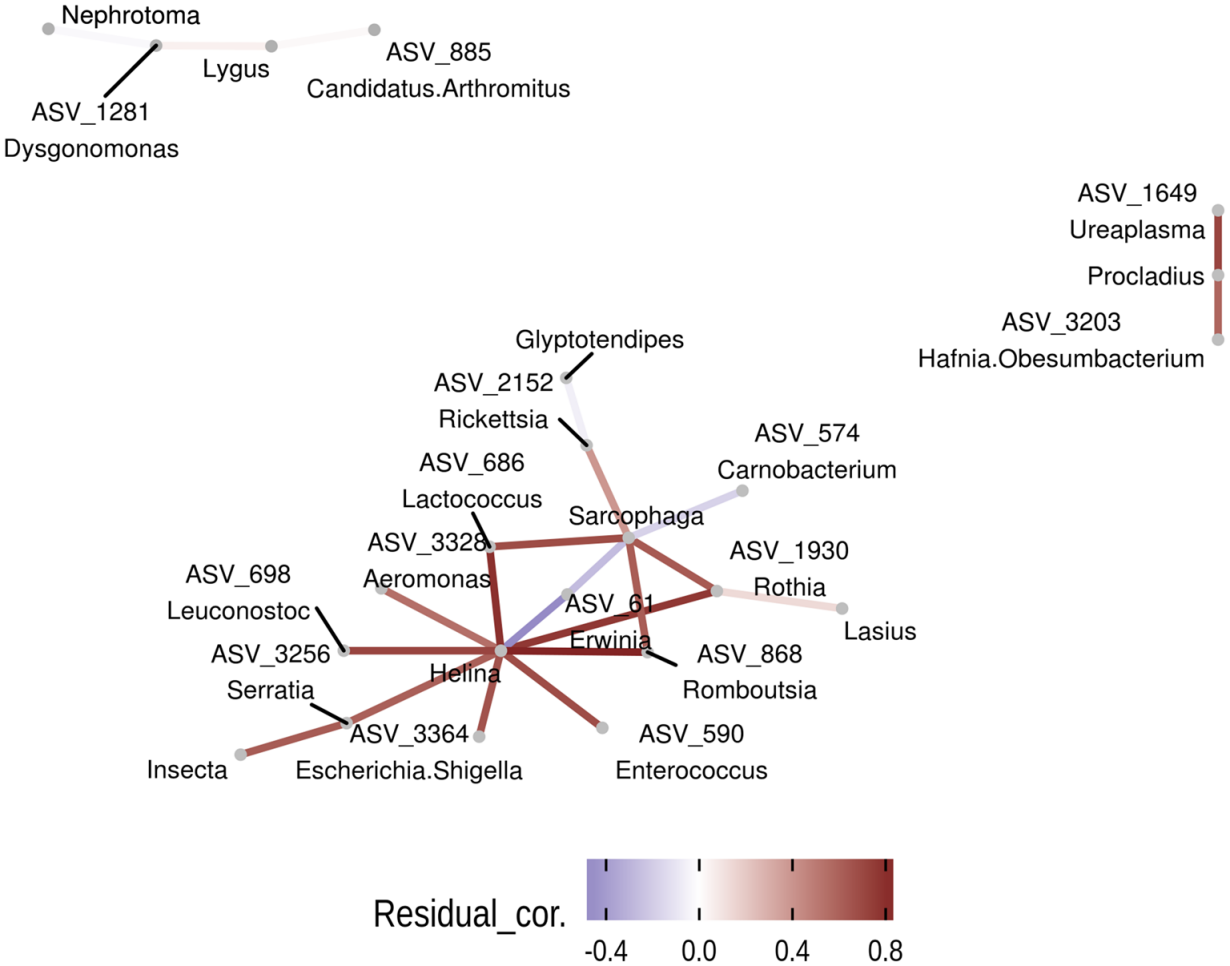
Residual correlations between bacterial ASVs and insect genera detected in faecal samples based on JSDM. Shown are correlations with posterior support > 0.95.

Bivariate Procrustean analysis revealed a significant congruence between dietary profiles and faecal microbiota or metagenome content composition with Procrustean correlation coefficients between 0.51 and 0.80 (Supporting information Fig. A5 and Table A3). Furthermore, db-RDA and subsequent variation partition analyses indicated that diet had a low, though significant, effect on faecal microbiota, independent of other covariates (R^2^_adjusted_ ranging between 0.033 and 0.149, Supporting information Table A4). Db-RDA models also revealed considerable effect of diet on predicted metagenome functions (Supporting information Table A4). Moreover, the most important principal components of dietary variation typically correlated with microbiota composition and its predicted functions (Supporting information Table A5). Faecal microbiota was also significantly affected by an independent effect of environmental covariates (R^2^_adjusted_ ranging between 0.022 and 0.048, Supporting information Table A4). Finally, variation partitioning revealed a fraction of faecal microbiota variation explained by both diet and environment (R^2^_adjusted_ ranging between 0.005 and 0.027, Supporting information Table A4). JSDM indicated 23 highly supported (posterior confidence > 0.95 for both fitted JSDM models) residual correlations between bacterial ASVs and insect genera present in the dietary profile (Fig. 4) and 14 highly supported residual correlations between insect genera and predicted metagenome pathways (Supporting information Fig. A6).

## Discussion

### Variation in barn swallow diet

Diptera represented the largest fraction of reads in our study, followed by Coleoptera, Hemiptera, and Hymenoptera. Interestingly, dietary profiles were mostly dominated by a single insect genus. We suspect this is due to the short retention and digestion time typical for birds^67,68^. The order-level taxonomic composition of diet in our population was roughly comparable to the dietary content reported in previous studies based on macroscopic examination of undigested food remains or faecal samples^28,69–72^. However, the dominance of individual insect groups tends to differ between previously published studies, presumably because of dietary variation in space and throughout the breeding season^28,69,71^. We explicitly addressed this possibility by field sampling within two months of the breeding season at two ca. 4.5 km distant breeding colonies, and were able to show that both spatial variation and collection date affected consumed diet. This illustrates the high spatio-temporal variability of dietary items consumed and shows that the dietary patterns observed during short sampling periods or at single locality cannot be easily generalised, even in aerial insectivores. The temporal variation in consumed diet can be related to the fact that the abundance peaks varied among consumed insects. Similarly, differences in diet composition between the two breeding colonies may be explained by differences in breeding livestock or other environmental factors. Unfortunately, we cannot directly support these explanations because prey availability was not quantified. Obtaining such data can be problematic with regards to aerial foragers, particularly as their hunting strategy, including intensity of hunting, height of hunting trips and their distance from the nest site, may vary dramatically with actual environmental conditions^72–74^.

Offspring can be very demanding with regards to the quality and quantity of nutrients required during the early post-hatching phases of development; hence, parents of many animal species supplement the offspring’s diet with specific dietary items^75,76^ or select breeding microhabitats that satisfy their dietary requirements^77,78^. Furthermore, adults may switch their typical foraging preferences during the breeding season in order to provide their progeny with a high-quality diet. Previous studies on the barn swallow suggest that parents feed themselves with smaller dietary items than those they provide to nestlings^72,79^. In the present study, however, both dietary composition and diet alpha diversity failed to provide support for the idea that food composition differs between adults and juveniles.

### Correlation between diet and faecal microbiota

While knowledge of gut microbiota in free-living vertebrates is gradually increasing, the extent to which their gut microbiota is affected by variation in diet is still not sufficiently understood. Most studies on wild vertebrate species have applied a comparative approach aimed at detection of microbiota variation between animal species^13,20,24,80^ or populations^18,81^. However, to our knowledge, there have been just a few studies attempting to directly integrate metabarcoding data on dietary composition and microbiota profiles on an individual basis^19,82–85^.

In comparison with more widely studied mammals, passerine birds have a clearly distinct composition of host-associated microbial communities^11,23,80^. The microbiota in such communities is characterised by rapid temporal changes at the intra-individual level, with just a few bacteria exhibiting some level of stability over time^41^. Based on current knowledge, interspecific differences in passerine gut microbiota composition appear to be rather low^23,80^. Furthermore, it has been shown that passerine gut microbiota structure can be affected by social contacts, age, sex, host immunity or blood concentrations of steroid hormones^43,86–88^. All the above-mentioned factors, however, usually explain just a limited fraction of total gastrointestinal microbiota variation. As such, we hypothesised whether the unexplained variation in gut microbiota may be related to actual diet composition. While bivariate Procrustean analysis indicated a significant correlation between gut microbiota and diet profile composition, use of this approach is problematic as bivariate approaches fail to distinguish direct links from indirect effects mediated by shared correlation of taxa abundance with environmental variables. To address this, we applied db-RDA modelling followed by variance partitioning, which indicated a significant fraction of gut microbiota variation explained by variation in diet, independent of the effect of environmental covariates modulating gut microbiota and/or diet consumed. Interestingly, the db-RDA models showed the strongest association between diet and phylogenetically controlled weighted UniFrac dissimilarities in microbiota composition and predicted metagenome functions. This suggests that individual insect taxa impose similar effects on related bacteria that likely have similar metabolic functions. JSDM identified 23 correlations between prey genera and bacterial ASVs. Most of these correlations were associated with abundance changes in *Helina* and *Sarcophaga* flies. In some cases, bacterial ASVs involved in these interactions corresponded to putative insect symbionts (e.g. a positive link between *Sarcophaga* and *Rickettsia* ASV_2152), suggesting that gut microbiota can be at least partly affected by bacteria present in the diet. At the same time, however, variation in dietary items was also associated with abundance changes in several bacteria that are widespread residents of vertebrate gut or reproductive tracts (e.g. *Candidatus* Arthromitus, *Enteroccocus*, *Ureaplasma*), suggesting that diet can also modulate proliferation of bacteria already residing in the barn swallow host. According to JSDMs, most changes in metagenome pathways were associated with abundance changes of *Sarcophaga* in diet*.* In particular, we observed positive correlations between *Sarcophaga* and several pathways involved in metabolism of nucleic acids and simple sugars. Despite being significant, the overall effect of diet on the gut microbiota of barn swallows, and in birds in general, appears to be of lower importance than in mammals^20^, providing further evidence for clear differences in host-microbiota interactions in these two vertebrate clades. Deducing mechanisms behind these differences is rather challenging, given the current state of knowledge. Nevertheless, we speculate that the explanation involves differences in digestion physiology between the two groups. In particular, diet passage through the gut is much faster in passerines than in mammals and, therefore, does not depend largely on bacterial fermentation^67,68^. Consequently, there would be a limited opportunity for bacterial populations within the gut to be affected by the diet consumed.

### Methodical considerations

In our study, we used recently designed universal COI primers that are comparable with existing primers for ribosomal genes in terms of their capability to target a wide range of arthropod taxa^45^. The broad taxonomic coverage achieved by our protocol was also evident based on our sequencing data, where several plant and fungal taxa were effectively amplified alongside barn swallow COI, resulting in a large proportion of non-target sequences in our dataset. Consequently, researchers intending to adopt these primers should account for this and adjust target sequencing depth accordingly. Further, to uncover potential biases in biological interpretations, researchers should also consider an in-depth missing values analysis, to identify sources of commonly occurring PCR failures and low numbers of target sequences.

To partly overcome the problem with non-target reads, primers blocking passerine COI amplification were added to the PCR reaction. Though this procedure is commonly used in metabarcoding-based diet analyses, it has been noted that blocking primers may systematically bias abundances of taxa in resulting profiles^89,90^. However, our data were unlikely to be affected as there was a high consistency in diversity and insect COI profile composition for sample duplicates that were prepared with and without blocking primers.

## Conclusions

Using COI profiling of faecal samples, we described diet variation in a breeding barn swallow population and demonstrated that diet metabarcoding is a promising non-invasive alternative to traditional diet analysis approaches in insectivorous birds. We also showed that use of blocking primers does not bias the content of diet profiles, probably due to phylogenetic disparity between passerines and their insect prey. The diet of barn swallows showed high interindividual variation, which was partly explained by variation among colonies and systematic changes during the breeding season. Finally, our data provides correlative support for the effect of diet consumed on faecal microbiota composition, independent of environmental factors affecting both diet and faecal microbiota.

## Supplementary Information

Supplementary Information 1.

Supplementary Information 2.

Supplementary Information 3.

## Data Availability

Sequencing data are available at the European Nucleotide Archive under project accession number PRJEB14586 for the 16S bacterial profile and PRJEB46476 for the COI profile. Accession numbers for each sample are provided in supporting information Table A1. Scripts associated with data analyses are archived in Github repository (https://github.com/jakubkreisinger/Swallow_diet).
