# Supplementary material for: Variation in diet composition and its relation to gut microbiota in a passerine bird
Source: Sci Rep. 2022 Mar 8;12:3787. doi: 10.1038/s41598-022-07672-9 (PMC8904835; doi:10.1038/s41598-022-07672-9)
Supplement: Supplementary file 1 — Supplementary Information 1. [file 41598_2022_7672_MOESM1_ESM.pdf]

Variation in diet composition and its relation to gut microbiota in a passerine bird

Lucie Schmiedová[1]*; Oldřich Tomášek[2]; Hana Pinkasová[1]; Tomáš Albrecht[1,2†]; Jakub Kreisinger[1†]

Figure A1: Taxa detected in barn swallow COI profiles for samples amplified using A) both gene specific and blocking primers or B) gene specific primers only.

A) both gene specific and blocking primers

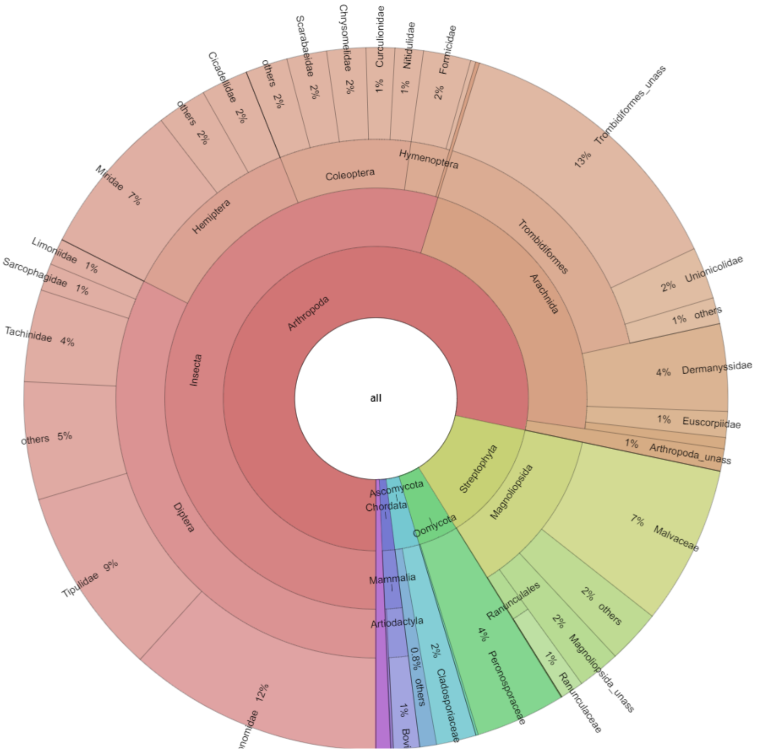

B) gene specific primers without the blocking primer

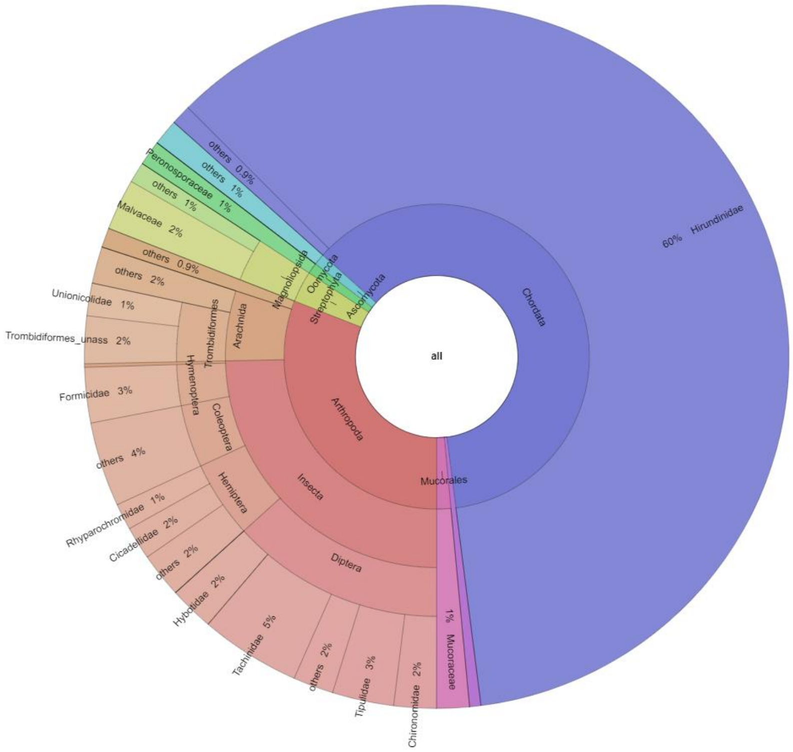

Figure A2: Insect taxa detected in barn swallow diet profiles.

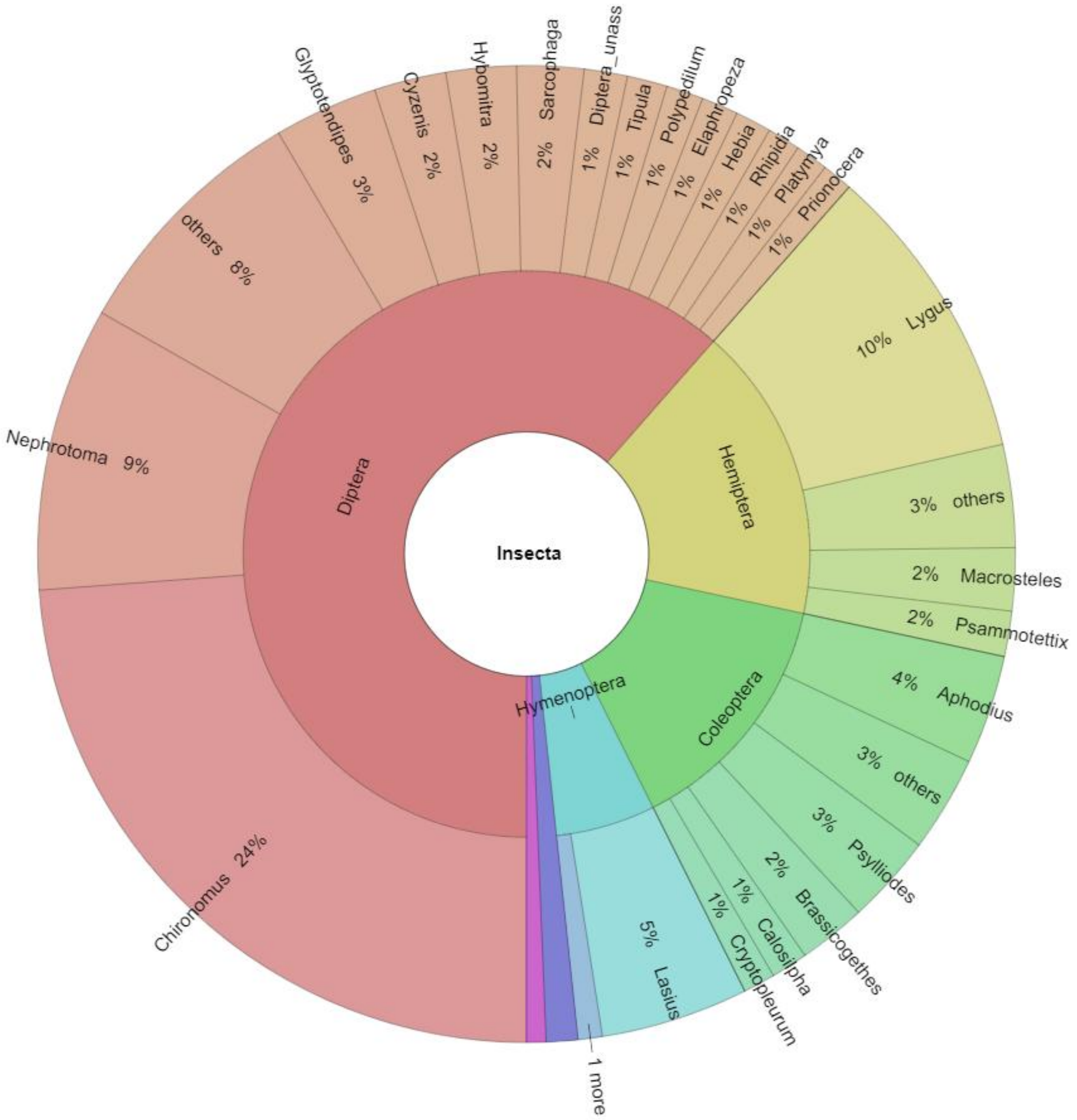

Figure A3: **Rarefaction curves for number of observed insect genera** within individual barn swallow samples. Curves were generated by 200 rounds of random subsampling of the origin community matrix. Lines indicate average values for given sequencing depths.

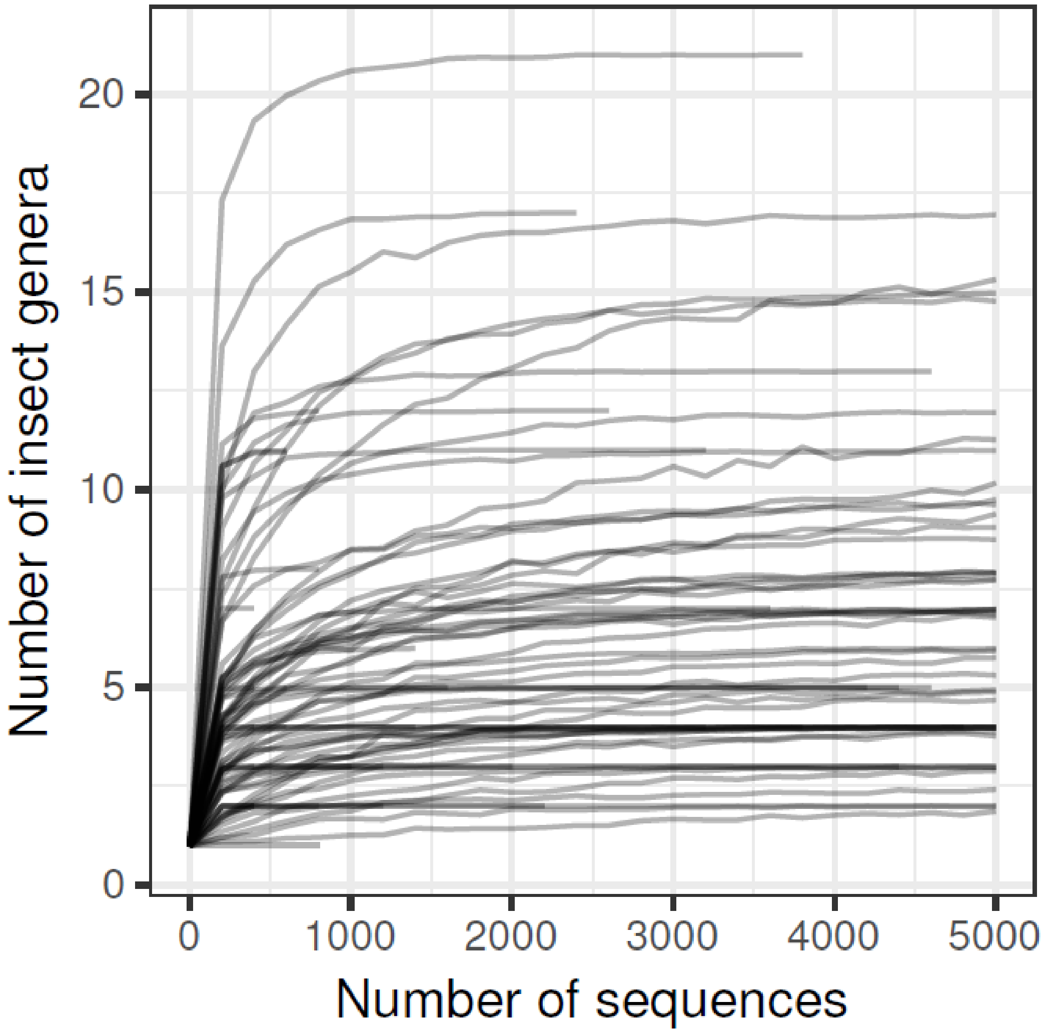

Figure A4: Temporal variation in the abundance of insect genera (expressed as read proportions). Shown are genera that exhibited significant abundance changes during the breeding season based on DESeq2 analysis. The lines and shaded areas correspond to the predictions and 95 % confidence intervals of the generalized linear models for data with negative binomial distribution fitted with the glmmTMB package. The Julian date was included as a second order polynomial. The model offset, which corresponds to the log-transformed number of sequences per sample was specified to account for uneven sequencing depth. Confidence intervals could not be calculated for *Coquillettidia*.

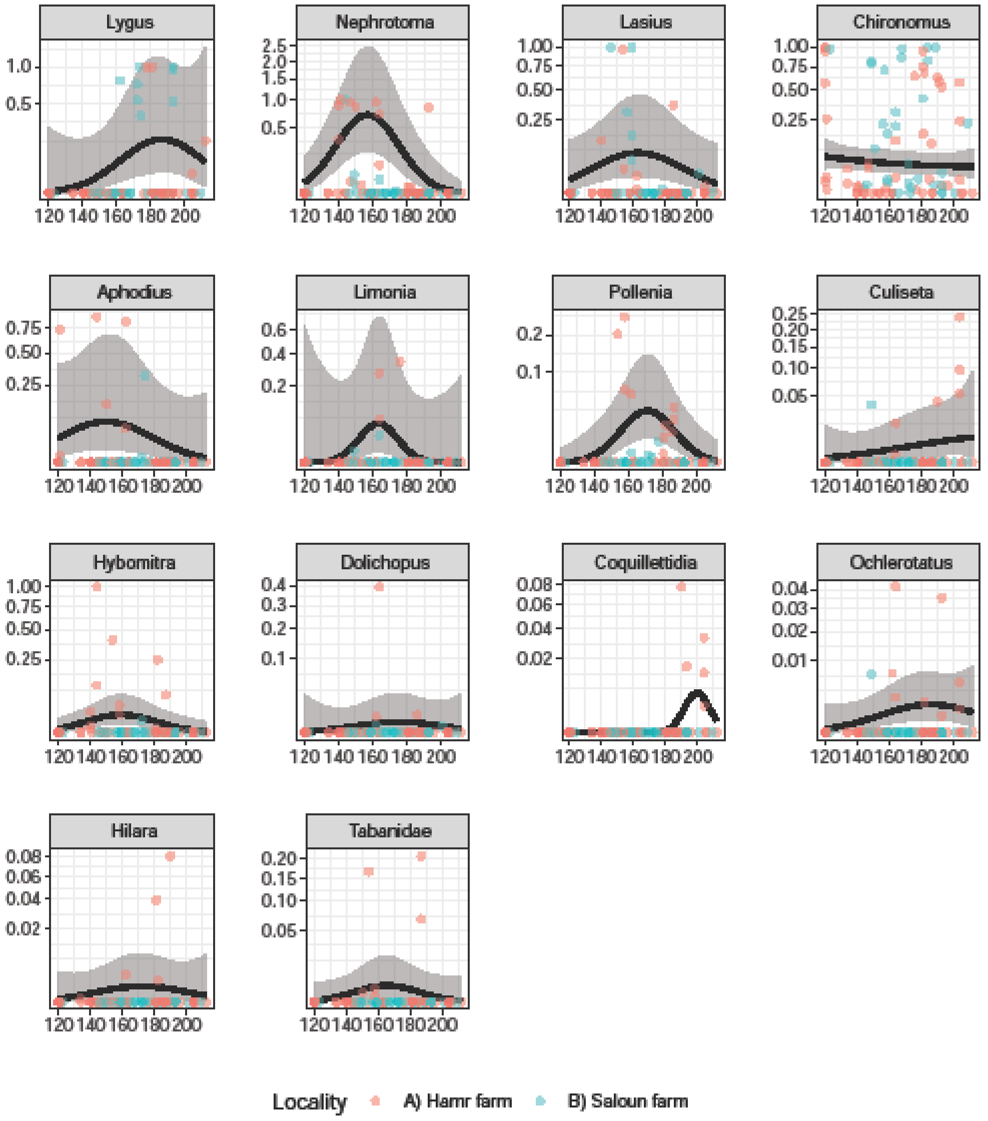

Figure A5: Procrustean superimposition for faecal microbiota vs. insect genera profiles.

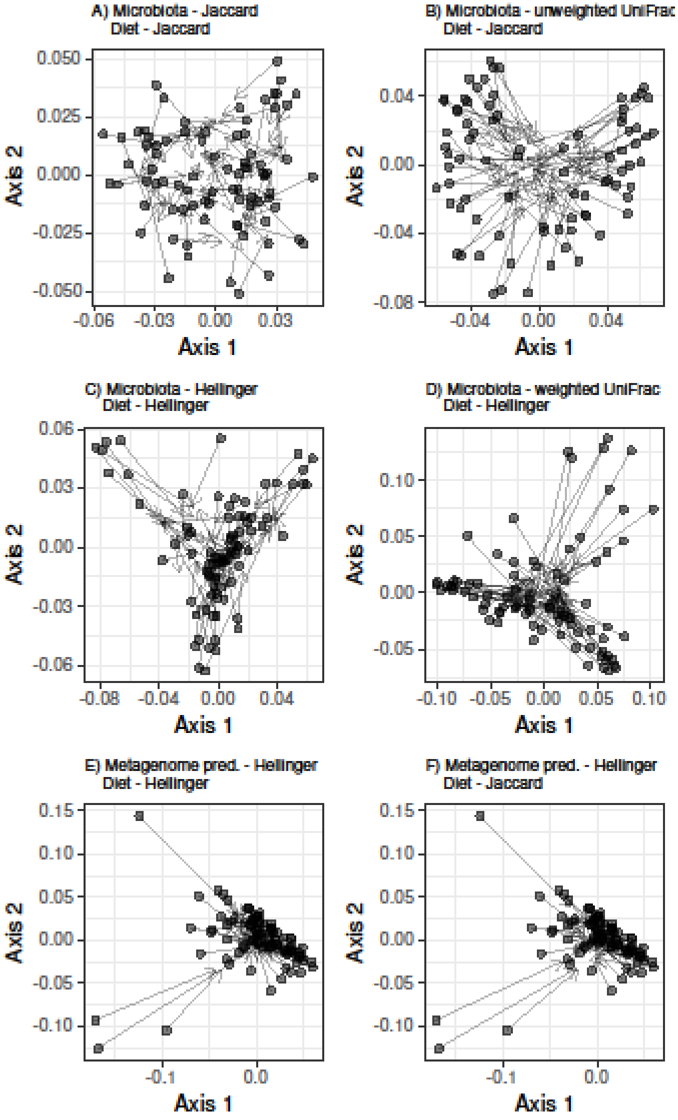

Figure A6: Residual correlations between predicted metagenome pathways and insect genera detected in faecal samples based on JSDM. Shown are correlations with consensual posterior support > 0.95.

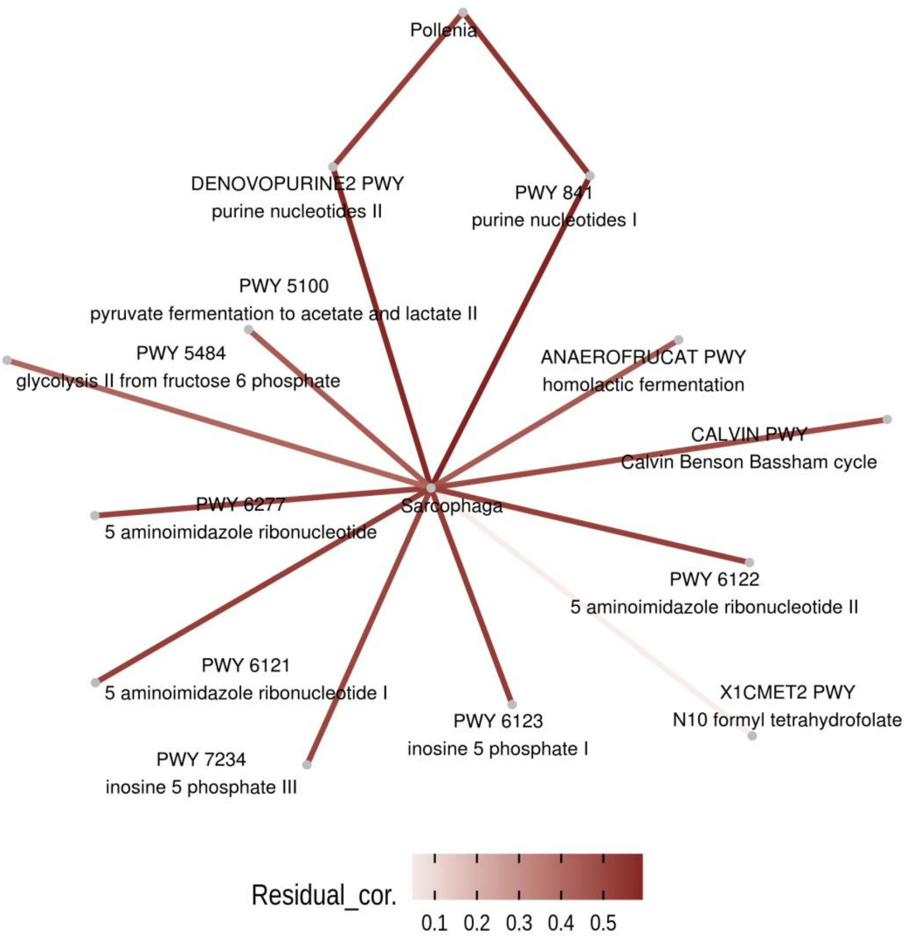

Table A3: Results of Procrustean analyses testing multivariate correlation between diet and gut microbiota composition or predicted gut microbiota functions. Analyses were performed on the PCoA axes for diet dissimilarities (Hellinger or Jaccard), microbiota dissimilarities (Hellinger, Jaccard, and weighted or unweighted UniFrac), or predicted metagenome pathways (Hellinger). Procrustean sums of squared differences, correlation coefficients and associated probability values are shown in the table.

Microbiota diss.	Diet diss.	Sum.of.squared.differences	Procrustes.correlation	p
Jaccard	Jaccard	0.3560	0.8025	0.001
unw. UniFrac	Jaccard	0.5573	0.6653	0.001
Hellinger	Hellinger	0.5525	0.6689	0.001
w. UniFrac	Hellinger	0.7357	0.5141	0.002
Hellinger (metagenome)	Hellinger	0.6619	0.5814	0.001
Hellinger (metagenome)	Jaccard	0.5856	0.6438	0.001

Table A4: Adjusted proportions of variance in gut microbiota composition or predicted metagenomes explained by the effect of diet, environmental predictors, or both. The number of PCoA axes for microbiota and other environmental predictors retained in the final db-RDA models is shown in the table.

Microbiota diss.	Diet diss.	no.axes diet	no.axes covariates	Diet	Coveriates	Both
Jaccard	Jaccard	10	5	0.0333	0.0219	0.0191
unw. UniFrac	Jaccard	4	5	0.0356	0.0478	0.0159
Hellinger	Hellinger	13	5	0.0627	0.0282	0.0274
w. UniFrac	Hellinger	11	5	0.1494	0.0468	0.0046
Hellinger (metagenome)	Hellinger	17	5	0.2785	0.0303	0.0088
Hellinger (metagenome)	Jaccard	13	5	0.1385	0.0076	0.0316

Table A5: Eigenvalues and relative eigenvalues (i.e., proportion of variation explained) of PCoA axes corresponding to dissimilarities in diet composition that were significantly associated with variation in microbiota according to db-RDA analyses.

Microbiota diss.	Diet diss.	Diet_PCOA_axes	Eigenvalues	Relative_eig
Jaccard	Jaccard	Axis.1	3.7966	0.0966
		Axis.2	2.3574	0.0611
		Axis.3	2.0537	0.0534
		Axis.4	1.6727	0.0438
		Axis.5	1.5243	0.0400
		Axis.8	1.1472	0.0303
		Axis.10	0.9564	0.0257
		Axis.12	0.8402	0.0228
		Axis.13	0.7996	0.0217
		Axis.25	0.4423	0.0128
unw. UniFrac	Jaccard	Axis.1	3.7966	0.0966
		Axis.2	2.3574	0.0611
		Axis.7	1.2757	0.0338
		Axis.8	1.1472	0.0303
Hellinger	Hellinger	Axis.1	13.4527	0.1943
		Axis.2	7.9994	0.1155
		Axis.4	3.6066	0.0521
		Axis.6	2.6473	0.0382
		Axis.8	2.1276	0.0307
		Axis.9	2.0886	0.0302
		Axis.10	1.8378	0.0265
		Axis.19	0.9762	0.0141
		Axis.30	0.6262	0.0090
		Axis.32	0.5673	0.0082
		Axis.45	0.0811	0.0012
		Axis.51	0.0419	0.0006
		Axis.68	0.0025	0.0000
w. UniFrac	Hellinger	Axis.1	13.4527	0.1943
		Axis.2	7.9994	0.1155
		Axis.8	2.1276	0.0307
		Axis.9	2.0886	0.0302
		Axis.10	1.8378	0.0265
		Axis.16	1.0677	0.0154
		Axis.22	0.9098	0.0131
		Axis.26	0.8081	0.0117
		Axis.38	0.2255	0.0033
		Axis.62	0.0076	0.0001
		Axis.81	0.0000	0.0000
Hellinger (metagenome)	Hellinger	Axis.2	7.9994	0.1155
		Axis.8	2.1276	0.0307

		Axis.9	2.0886	0.0302
		Axis.11	1.7604	0.0254
		Axis.13	1.3761	0.0199
		Axis.19	0.9762	0.0141
		Axis.21	0.9453	0.0137
		Axis.23	0.8701	0.0126
		Axis.24	0.8535	0.0123
		Axis.25	0.8463	0.0122
		Axis.29	0.6672	0.0096
		Axis.41	0.1502	0.0022
		Axis.42	0.1041	0.0015
		Axis.47	0.0718	0.0010
		Axis.51	0.0419	0.0006
		Axis.53	0.0306	0.0004
		Axis.54	0.0256	0.0004
Hellinger (metagenome)	Jaccard	Axis.2	2.3574	0.0611
		Axis.8	1.1472	0.0303
		Axis.12	0.8402	0.0228
		Axis.15	0.7053	0.0194
		Axis.16	0.6740	0.0185
		Axis.20	0.5282	0.0150
		Axis.21	0.5102	0.0145
		Axis.23	0.4817	0.0138
		Axis.25	0.4423	0.0128
		Axis.32	0.3217	0.0098
		Axis.34	0.2804	0.0088
		Axis.38	0.2464	0.0079
		Axis.42	0.2148	0.0072
